# Differential Characterization of Temozolomide-Resistant Human Glioma Cells

**DOI:** 10.3390/ijms19010127

**Published:** 2018-01-02

**Authors:** Sheng-Wei Lai, Bor-Ren Huang, Yu-Shu Liu, Hsiao-Yun Lin, Chun-Chuan Chen, Cheng-Fang Tsai, Dah-Yuu Lu, Chingju Lin

**Affiliations:** 1 Graduate Institute of Basic Medical Science, China Medical University, Taichung 40402, Taiwan; wayson081024@gmail.com; 2 Graduate Institute of Clinical Medical Science, China Medical University, Taichung 40402, Taiwan; bluemoon1201.tw@yahoo.com.tw; 3 Neurosurgery Department, Taichung Tzu Chi Hospital, Buddhist Tzu Chi Medical Foundation, Taichung 42743, Taiwan; 4 School of Medicine, Tzu Chi University, Hualien 97002, Taiwan; 5 Department of Biotechnology, Asia University, Taichung 41354, Taiwan; yushuliu220@gmail.com (Y.-S.L.); tsaicf@asia.edu.tw (C.-F.T.); 6 Department of Pharmacology, School of Medicine, China Medical University, Taichung 40402, Taiwan; lingirl831@hotmail.com; 7 Graduate Institute of Biochemistry, National Chung Hsing University, Taichung 40249, Taiwan; yober86@hotmail.com; 8 Department of Photonics and Communication Engineering, Asia University, Taichung 41354, Taiwan; 9 Department of Physiology, School of Medicine, China Medical University, Taichung 40402, Taiwan

**Keywords:** glioblastoma, temozolomide, connexin 43, drug-resistant

## Abstract

Glioblastoma multiforme (GBM) is the most common type of primary and malignant tumor occurring in the adult central nervous system. Temozolomide (TMZ) has been considered to be one of the most effective chemotherapeutic agents to prolong the survival of patients with glioblastoma. Many glioma cells develop drug-resistance against TMZ that is mediated by increasing *O*-6-methylguanine-DNA methyltransferase (MGMT) levels. The expression of connexin 43 was increased in the resistant U251 subline compared with the parental U251 cells. The expression of epithelial–mesenchymal transition (EMT)-associated regulators, including vimentin, N-cadherin, and β-catenin, was reduced in the resistant U251 subline. In addition, the resistant U251 subline exhibited decreased cell migratory activity and monocyte adhesion ability compared to the parental U251 cells. Furthermore, the resistant U251 subline also expressed lower levels of vascular cell adhesion molecule (VCAM)-1 after treatment with recombinant tumor necrosis factor (TNF)-α. These findings suggest differential characteristics in the drug-resistant GBM from the parental glioma cells.

## 1. Introduction

Glioblastoma multiforme (GBM) is the most common primary brain tumor that affects the central nervous system in adults and is one of the deadliest cancers in humans [1]. GBM is characterized by rapid growth, becoming resistant to conventional treatments and poor prognosis [2]. The current clinical management of GBM includes surgical resection combined with radiotherapy and chemotherapy, among which is the treatment of the DNA alkylating agent temozolomide (TMZ) [3], which readily penetrates the blood–brain barrier (BBB). Unfortunately, even with combined treatments, the survival rate hovers around 25% and most patients die within 2 years after the diagnosis [3,4]. A putative markers of glioma progression is the gap junctions (GJs) that highly distributed in the brain [5]. In glioma cells, gap junctions are important channels that contribute to cell-to-cell communication by allowing intercellular transportation of small molecules, second messengers, ions, and electrical signals that help to maintain homeostasis in the multicellular system [6]. Connexin 43 (Cx43), also called gap junction alpha-1 protein (GJA1), is the major component protein of gap junctions in astrocytes [7]. Recently, a number of groups have reported several lines of evidences that the gap junction protein Cx43 regulates the responses of GBM cells to TMZ [8]. A preclinical study has observed an inverse correlation between the Cx43 protein levels in patients and TMZ sensitivity of GBM cells, including GBM stem cells [9]. The study stated that the expression level of Cx43 was inversely correlated with a patient’s survival rate. Some studies have shown that Cx43 is downregulated in high-grade brain tumors [10,11], while others have demonstrated that an increased level of Cx43 is capable of conferring chemotherapeutic resistance to human glioma cells through the upregulation molecular mechanisms [12,13]. Overall, this reflects the lack of consensus for the pro- or anti-tumorigenic role for connexins in GBM.

The invasion of glioma cells into adjacent brain tissues occur in accordance with the activation of multigenic programs. The tumor cell growth and invasion is linked to epithelial–mesenchymal transition (EMT), which is the most important biological process in gliomagenesis and cancer progression [14,15]. EMT is an essential mechanism for the transformation of local glioma tumors into infiltrating tumors by a switching process, where polarized epithelial cells transform into motile mesenchymal cells [16,17]. Recent emerging evidences demonstrated a molecular and phenotypic association between increased chemoresistance and gaining of EMT-like phenotype of cancer cells. EMT also leads to the resistance of glioma cells against various therapeutic strategies [18]. In this study, we evaluated differences between parental glioma cells and a selected resistance subline with respect to migration abilities.

Cancer invasion is complex and involves integrated biochemical processes requiring coordinated efforts of intracellular and extracellular interactions [19]. Therefore, cell surface adhesion molecules play important roles in the cell–cell or cell–microenvironment interactions. It has been reported that the cytokine, TNF-α, is capable of inducing the upregulation of vascular cell adhesion molecule-1 (VCAM-1) and intracellular adhesion molecule-1 (ICAM-1) [20,21]. VCAM-1 is a molecule with a well-characterized role in the human immune system. It has been shown to be expressed on the surface of tumors and interact with cells that promote tumor invasion, angiogenesis, and metastasis [22]. There are evidences indicating that VCAM-1 is upregulated in breast cancer cells and the VCAM-1 signaling networks involves in controlling EMT and chemoresistance in malignant breast tumors [23]. Several recent studies have investigated the mechanisms of tumor escape from immune surveillance and attack that may blunt the efficacious effects of active immunotherapy [24,25]. These studies have identified many distinct loss-of-function and gain-of-function mechanisms of tumor immune evasion. The findings suggested that expression of VCAM-1 in tumors promoted T-cell migration away from tumors, resulting in decreased accumulation of T cells in the tumor microenvironment. This decreased accumulation of T cells around tumor cells may contribute to the ability of VCAM-1 expressing tumor cells to escape immune attack [26]. Another publication demonstrated that high expression of VCAM-1 correlates with abundant monocyte/macrophage infiltration in tumors [27,28]. Thus, we investigated whether there were differential responses between parental glioma cells and the selected TMZ-resistance subline cells under the recombinant TNF-α stimulation.

In this study, protein expression revealed that higher levels of MGMT and Cx43 correlated with TMZ-resistance in glioma cells. We selected the U251 migration-prone subline and characterized its properties with parental glioma cells with respect to migration abilities and the expression of related molecules [29]. Our previous study revealed that higher levels of VCAM-1 correlated with poor prognosis in glioma patients. Besides, the study also showed that EGFR-dependent VCAM-1 expression promoted human monocyte adhesion to GBM [30]. In this study, we investigated and revealed a differential expression of VCAM-1 between the TMZ-resistant and the parental glioma cells.

## 2. Results

### 2.1. MGMT and Cx43 Are Upregulated in the TMZ-Resistant U251 Cells

**Figure 1:**
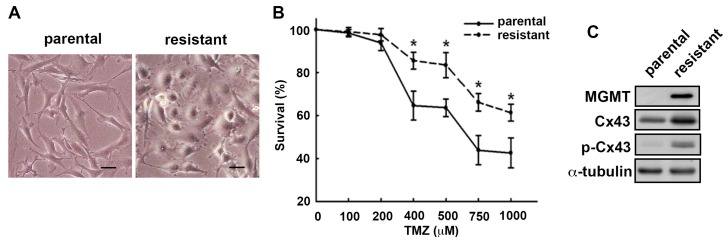
Effects of TMZ treatment on MGMT and Cx43 expression in GBM. Human U251 glioma cells were treated with TMZ or non-treated to induce TMZ-resistant U251 cells and parental U251 cells, respectively. (**A**) The TMZ-resistant cells and the parental cells have a distinctive morphology. Scale bar = 20 μM. (**B**) Determination of the TMZ (0–1000 µM, for 72 h)-induced cell death in parental U251 cells and TMZ-resistant U251 cells. Analyzed by SRB assay for cell viability. Quantitative data are presented as mean ± SEM of three independent experiments. * *p* < 0.05 compared with the parental group; (**C**) MGMT, Cx43, and p-Cx43 expression were determined using Western blot analysis in parental and TMZ-resistant cells.

We first characterized the cells based on their morphology. Specifically, the TMZ-resistant cells appeared epithelial-like morphology and are polygonal or round in shape, whereas the parental cells assumed a fibroblastic-like morphology with elongated phenotypes (Figure 1A). We further evaluated the survival rates in TMZ-induced cell death between the parental cells and the TMZ-resistant cells. The cytotoxicity of TMZ ranging from 0 to 1000 μM for 72 h treatment was determined by analyzing cell viability using SRB assay (Figure 1B). The TMZ-resistant subline showed a higher survival rate upon the TMZ treatment than that of the parental cells. TMZ resistance is often associated with an increased MGMT expression, an enzyme that repairs DNA damage induced by TMZ. In addition, it has been reported that Cx43 was found to be more abundantly expressed in GBM tissues when compared with normal brains. Overexpressing Cx43 in GBM cell lines rendered them resistant to TMZ [12,13]. To correlate the relationship between Cx43 and the TMZ resistance, we examined MGMT and Cx43 protein expressions in the U251 parental and TMZ-resistant cells. Consistent with previous reports, the results revealed that MGMT, Cx43, and the phosphorylated Cx43 protein expressions were all upregulated in the TMZ-resistant cells (Figure 1C). The mRNA expressions of MGMT and Cx43 of the TMZ-resistant cells were approximately 300- and 150-fold higher than those of the parental cells.

### 2.2. The TMZ-Resistant Subline and the Parental Cells Exhibited Differential Migratory Abilities and Protein Expression Profiles

**Figure 2:**
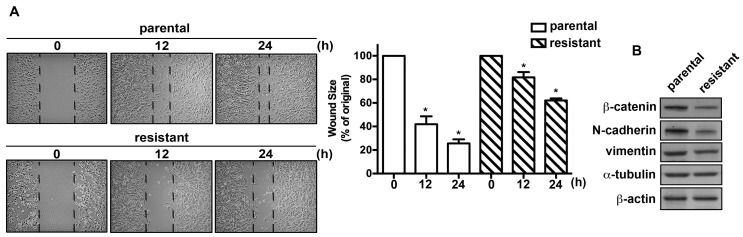
TMZ-resistant cells exhibited lower migratory ability than parental glioma cells. (**A**) After the TMZ selection, the parental U251 and their corresponding TMZ-resistant subline were seeded for indicated time periods (0, 12, and 24 h). Cell migration was determined using a wound-healing assay. TMZ-resistant cells exhibited decreased migration ability compared with parental cells. Representative images are shown. Quantitative data are presented as mean ± SEM of three independent experiments. * *p* < 0.05 compared with the control group. (**B**) The protein expression profiles of the U251 and the TMZ-resistant cells. Protein expression levels of EMT-associated markers were determined using Western blotting.

We next evaluated the migratory activities of the U251 and the TMZ-resistant cells. The TMZ-resistant cells exhibited reduced healing ability compared with the parental cells (Figure 2A). TMZ-resistant cells also showed decreased cell mobility and migrated less readily through the membrane pores of cell culture inserts compared with the parental U251 cells. The mobility of the resistant subline cells was approximately 2- and 3-fold lower than that of the parental cells (Figure 2A). Moreover, protein analysis showed that the expression levels of EMT invasive-associated molecules, including β-catenin, N-cadherin, and vimentin, were lower in TMZ-resistant cells than those of the parental cells (Figure 2B). We also examined the proliferation rates between the TMZ-resistant cells and the parental cells. However, no significant differences of cell proliferation between these two cells lines were observed.

### 2.3. The TMZ-Resistant Subline Showed Decreased Monocyte Adhesion Ability and the Differential Expression of Proliferation-Related Proteins

**Figure 3:**
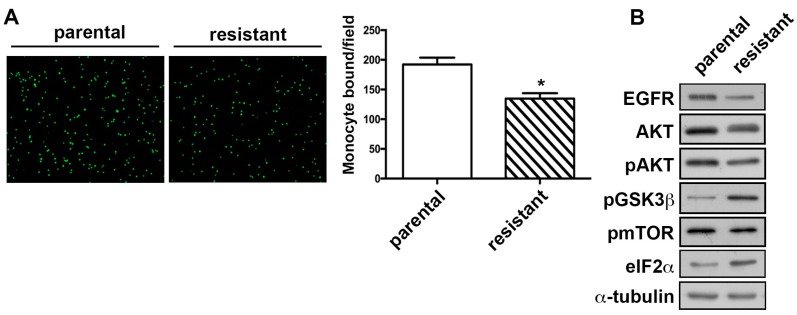
TMZ-resistant cells exhibited lower monocyte adhesion ability than the parental glioma cells. (**A**) Parental and TMZ-resistant cells were seeded for 24 h. Followed by incubation with the addition of BCECF-AM-labeled-THP-1 for 30 min, the adherence of THP-1 to GBM was evaluated. The ability of monocyte adhesion to GBM was evaluated by measuring the number of BCECFAM-labeled-THP-1 by the fluorescence microscopy. Quantitative data are presented as mean ± SEM of three independent experiments. * *p* < 0.05 compared with the parental group. (**B**) The protein expression profiles of parental and TMZ-resistant cells. Protein expression levels of proliferation-associated markers were determined using Western blotting.

Several studies reported that the monocytes/macrophages are the major glioma-associated inflammatory cells that constituted the tumor microenvironment [31]. Importantly, a recent report and a clinical study revealed that those monocytes/macrophages are the most predominant tumor-associated macrophages (TAMs) in GBM [32,33]. It has been indicated that suppressing the tumor-promoting effects of monocytes in glioma could be considered as an adjuvant treatment [34]. The ability of monocytes binding to GBM was determined by the monocyte-binding assay. We compared the monocyte adhesion ability between the TMZ-resistant subline and the parental cells, and it revealed that the TMZ-resistant subline exhibited reduced monocyte adhesion compared with the parental cells (Figure 3A). The binding of epidermal growth factor (EGF) to its receptor (EGFR) activates several signaling intermediates, including AKT, leading to control of cell survival and metabolism [35]. We further investigated the expression levels of proliferation-associated molecules and found that the expressions of EGFR and AKT were decreased in TMZ-resistant cells (Figure 3B). In addition, it has been reported that the activation of AKT leads to activate kinases and inhibit GSK3 by phosphorylating the inhibitory serines on GSK3 in resting cells [36]. The phosphorylation level of GSK3β can be enhanced by the activation of eIF2α kinases [37]. According to our data, elevated levels of phosphorylated GSK3β and eIF2α expression were observed in TMZ-resistant cells (Figure 3B).

### 2.4. The TMZ-Resistant Subline Exhibited Lower Sensitivity to TNFα-Induction

**Figure 4:**
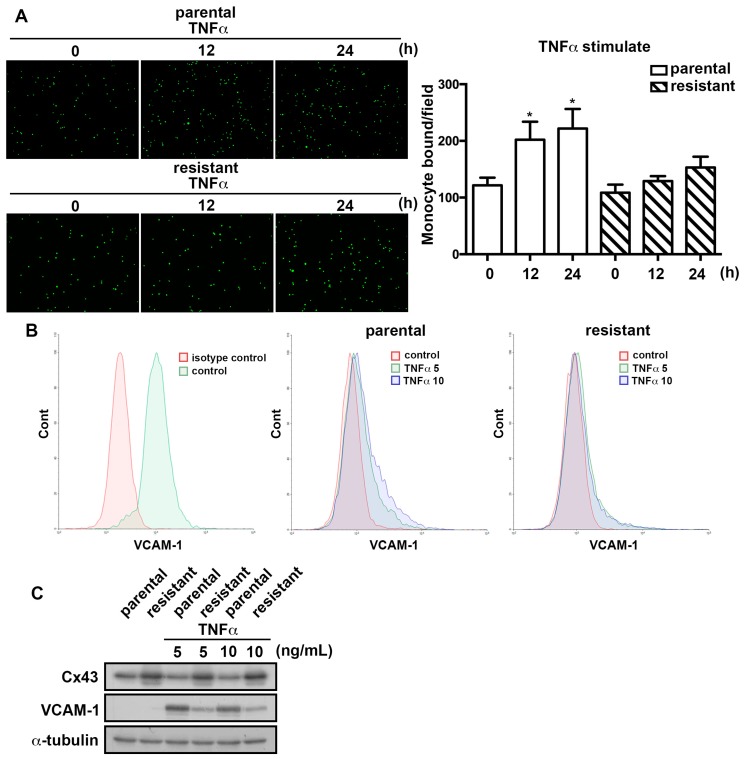
TMZ-resistant cells exhibited a lower sensitivity to TNF-α-induced VCAM-1 expression. (**A**) Parental U251 and TMZ-resistant cells were treated with TNF-α (10 ng/mL) for indicated time periods (0, 12, 24 h) followed by incubation with BCECF-AM-labeled-THP-1 for 30 min. The monocyte-binding ability was examined by the fluorescence microscopy and the one-way ANOVA analysis of variance was performed. The data are presented as mean ± SEM of three independent experiments. * *p* < 0.05 compared with the control group. (**B**) U251 and TMZ-resistant cells were treated with various concentrations of TNF-α (5 or 10 ng/mL) for 24 h and the expression of cell surface VCAM-1 was determined by using flow cytometry. (**C**) Cells were treated with various concentrations (5 or 10 ng/mL) of TNF-α for 24 h. VCAM-1 and Cx43 expressions were determined using Western blot analysis.

TNF-α is a major cytokine in the tumor microenvironment and its expression correlates with the GBM tumor grades [38,39]. We next examined the effect of TNF-α on monocyte adhesion in GBM. As shown in Figure 4A, treatment of GBMs with TNF-α induced THP-1 monocyte adhesion to GBM in a time-dependent manner. Interestingly, TNF-α treatment was found to depress monocyte adhesion ability in the TMZ-resistant cells compared with the parental cells. We next evaluated the effects of the cytokine administration on the induction of VCAM-1 expression. The flow cytometry analysis revealed that the expression of VCAM-1 was elevated by the TNF-α treatment in the parental cells. However, the expression of VCAM-1 induced by TNF-α was decreased in the TMZ-resistant cells (Figure 4B). The same results were also observed by Western blot analysis (Figure 4C). These findings suggest that the TMZ-resistant subline had lower sensitivity to TNF-α-induced monocyte adhesion and VCAM-1 expression than U251 parental cells.

## 3. Discussion

The present study demonstrated the heterogeneous nature of GBM cells and the inherent issues of treating any CNS cancer due to its limited repair mechanisms that make a presumably successful treatment of drug resistant GBM cells a difficult goal [40]. In this study, we have observed several characteristics of the TMZ-resistant cells. First, we established a cancer cell subline with high TMZ-resistant ability and revealed that the TMZ-resistant subline had lower migration and invasion ability. Second, we found more abundant expression of MGMT in TMZ-resistant cells, which also showed higher expression levels of gap junction protein Cx43. During cancer progression, the tumor cell migration is essential for invasion and dissemination into surrounding tissues [41]. Clinical conditions where epithelial cells are transformed to a mesenchymal phenotype usually indicate poor prognostic outcome [42]. Third, the results showed that the TMZ-resistant subline has decreased migratory activity compared with the parental cells. The differential expression levels of EMT-associated markers were observed between these two cell lines. Fourth, the TMZ-resistant cells were less liable than the parental cells to TNF-α treatment with regard to the expression of VCAM-1 and monocyte adhesion.

In clinical studies, even patients with initial glioma tumor control will inevitably relapse or progress during or after TMZ therapy. In human gliomas, the expression of MGMT proteins has been detected and regarded as a prognostic factor [43,44]. Low basal expressions of the tumor MGMT protein and the MGMT promoter hyper-methylation are each associated with prolonged survival for GBM patients treated with TMZ [45,46]. A previous study has shown that the protein expression of MGMT could serve as a potential negative molecular regulator of GBM migration and invasion [47].

There is a general agreement that the heterocellular Cx43 gap junctions formed between glioma-astrocytes and glioma-endothelial cells facilitate invasion [48,49,50]. Cx43 can exert both tumor suppression and oncogenic functions [51]. During epithelial-to-mesenchymal transition and the period of overt tumor infiltration, there is a loss of intercellular communication through gap junction (GJIC) between the malignant cells [52]. Furthermore, a reduction in Cx43 level in U251 human glioma cells usually showed an increase in migration [53,54]. Our results corroborated the results of a previous study indicating that Cx43 expression upregulated in the U251-resistant subline changes the mode of migration [55].

Emerging evidences have implicated that EMT is associated with cancer cell invasion and tumor progression. The EMT features correlate with poor survival rates and an increased risk of cancer recurrence among patients with various solid tumor types, such as colon cancer and breast cancer [56,57]. More importantly, EMT is also associated with therapeutic resistance [58]. Recent studies have shown that EMT was closely related to chemoresistance with elevated MGMT expression [59]. Therefore, canonical signaling regulated by MAPK and PI3K/AKT have involved in the regulation of EMT concerning the tumor resistance and recurrence [58]. Taken together, cancer progression mechanisms are intimately linked to EMT in multiple solid tumors. Thus, the role of EMT in therapeutic resistance is evident. This is the result both of the stem cell-like properties acquired by cells undergoing EMT and of its being a means of potentiating tumor heterogeneity. Our study investigated the role of EMT in drug-resistance and heterogeneity of these different lines in terms of tumor control of cell motility. While many studies have shown that migration and EMT are positively correlated with high grade and resistant phenotype in glioma cells, our study implies that other mechanisms might be involved in EMT’s negatively regulating the motility of TMZ-resistant cells.

EGFR is most commonly amplified and mutated in GBM, leading to overexpression and activation of downstream signaling pathways [60,61]. EGFR signals through a complex network of intermediates including PI3K, AKT, and MAPK. Overexpression of EGFR signaling results in proliferation, invasiveness, increased motility, inhibition of apoptosis, and resistance to chemotherapy [62]. Our previous study provided evidences that cytokines enhance VCAM-1 expression and monocyte adhesion, which is regulated by EGFR expression [30]. It has also been published that HER2/PI3K/AKT activation leads to multiple drug resistance in human breast cancer [63]. AKT pathway plays a role in conferring a broad chemoresistance in breast cancer and provides potential strategies for targeting this pathway for enhancing cancer chemotherapy [64]. The AKT targeting of mTOR signaling pathway is hyper-activated or altered in many cancer types and regulates a broad range of cellular processes including survival, angiogenesis, and metastasis [65]. However, no significant expression differences of phosphorylated mTOR were observed between the TMZ-resistant cells and the parental cells in this study. The best-characterized AKT targets are the GSK-3. In the current study, we showed the differential expression of the downstream GSK-3β and elF2α in glioma cell lines. An increased level of eIF2α kinases in the TMZ-resistant subline was observed, which conformed with a previous report that activated eIF2α kinases also leads to the activation of GSK3β [66]. 

Glioma-associated microglia has been reported to secrete immunosuppressive factors that contribute to immune surveillance [67]. Previous reports showed that the VCAM-1 expression in tumor cells has been considered as a potential therapeutic target in immune evasion [68]. Several recent studies have revealed the mechanisms of tumor escape from immune surveillance and attack that may blunt the efficacious effects of active immunotherapy [24,25]. The present study demonstrated that VCAM-1 expression was induced in GBM treated with TNF-α, which subsequently promoted the interaction between monocytes and GBM. Importantly, there was a positive correlation between the VCAM-1 expression and the activities of adherent monocytes in GBM [30]. Our previous study indicated that the levels of VCAM-1 in the GBM correlate highly with the activities of monocyte binding to GBM and that knockdown of VCAM-1 abolishes the enhancement of monocyte adhesion [30]. Our results showed that the TMZ-resistant subline had lower levels of TNF-α-induced VCAM-1 than that of the parental U251 cells, which may benefit the tumor microenvironment by achieving immune evasion. 

The complicated heterogeneity of glioblastoma multiforme attributes to the differential responses of different GBM sublines. This study identified functional differences between the U251 TMZ-resistant subline and the U251 parental cells. Although results from these cell models might not completely correlate with the clinic-pathological grades of glioma tissues, it is worth noting the distinct characteristics between the parental and the resistant sublines. In summary, our results show that connexin 43 was upregulated in the resistant U251 subline compared with the parental U251 cell. In addition, EMT-associated regulators were downregulated in the resistant U251 subline. The resistant U251 subline also exhibited lower cell migration and monocyte adhesion ability compared with the parental U251 cell. Furthermore, the resistant U251 subline also expressed lower levels of vascular cell adhesion molecule VCAM-1 than the parental cells upon stimulation with recombinant TNFα. These findings suggest differential characteristics in the drug-resistant GBM from parental glioma cells. These observations demonstrated that the extent of intratumoral heterogeneity in glioma influences cell migratory activity as well as the expression of intrinsic adhesion proteins. Further functional studies on potential molecular markers may provide therapeutic strategies for targeting malignant glioma cells.

## 4. Materials and Methods

### 4.1. Materials

TNF-α was purchased from PeproTech (Rocky Hill, NJ, USA). Primary antibodies specific for α-tubulin (T5168) and vimentin (V6630) were purchased from Sigma-Aldrich (St. Louis, MO, USA). Primary antibodies specific for VCAM-1 (ab134047), MGMT (ab108630), and β-catenin (ab16051) were purchased from Abcam (Cambridge, UK). Primary antibodies specific for AKT1/2/3 (sc-8312), p-AKT1/2/3 (Ser^473^, sc-7985-R), connexins 43 (sc-271837), eIF2α (sc-133132), and N-cadherin (sc-7939) were purchased from Santa Cruz Biotechnology (Santa Cruz, CA, USA). Primary antibodies specific for EGFR (4267), p-Cx43 (Ser^368^, 3511), and p-mTOR (Ser^2448^, 2971) were purchased from Cell Signaling Technology (Danvers, MA, USA). Primary antibodies specific for p-GSK3β (Tyr^216^, 44604G) was purchased from Invitrogen (Carlsbad, CA, USA).

### 4.2. Cell Culture

U251 human GBM cells were obtained from the Japanese Collection of Research Bioresources Cell Bank (JCRB No. IFO50288, Tokyo, Japan). THP-1 human monocytes were obtained from the Bioresource Collection and Research Center (BCRC Nos. 60360, 60582 and 60430; Hsinchu, Taiwan). TMZ-resistant subline were established and maintained in our laboratory. Briefly, the TMZ-resistant (U251-R) subline was generated by repetitive pulse exposure of U251 GBM cells to TMZ (500 µM) for 72 h every week. The undead U251-R cells were seeded at 6 cm dish and growing for 2 weeks. The cycle was repeated for 3 months. Multiple clones of U251-R were established to stably exhibit TMZ resistance. U251 were maintained in Minimum Essential Medium, THP-1 was maintained in RPMI-1640. All cells were cultured in medium supplemented with 10% fetal bovine serum, 100 U/mL penicillin, and 100 mg/mL streptomycin and were incubated at 37 °C in a humidified atmosphere containing 5% CO_2_ and 95% air.

### 4.3. Monocyte-Binding Assay

THP-1 cells were labeled with 0.1 μg/mL BCECF/AM (Invitrogen, Green) at 37 °C for 1 h followed by washed twice with growth medium. GBM cells were treated with TNF-α for indicated periods. The medium was removed from the wells, and 2.5 × 10^5^ BCECF/AM-labeled-THP-1 cells were added to a monolayer GBM cells. After incubation at 37 °C for 30 min, the wells were gently washed twice with warm growth medium to remove non-adherent cells. The cells were then photographed under a fluorescence microscope to calculate adherent cells.

### 4.4. Flow Cytometry Assay

GBM cells were incubated with anti-VCAM-1-conjugated PE antibody (eBioscience, 12-1069), isotype IgG control (eBioscience, San Diego, CA, USA) for 60 min at 4 °C. Expression of these surface receptors was determined using a NovoCyte flow cytometer (ACEA Biosciences, San Diego, CA, USA). Ten thousand events were recorded and GBM cells were examined for VCAM-1 expression.

### 4.5. Western Blot

The procedure of Western blotting was described previously [69,70]. In brief, the supernatants were collected by centrifugation at 13,000× *g* for 30 min and stored at −20 °C. Protein samples were separated by SDS-PAGE (sodium dodecyl sulfate-polyacrylamide) and transferred to nitrocellulose membranes. The membranes were blocked with 5% nonfat milk in Tris-Buffered Saline and Tween 20 (TBST) for 1 h at room temperature and then probed overnight with primary antibodies at 4 °C. After three TBST washes, the membranes were incubated with secondary antibodies. The blots were visualized using enhanced chemilumminescence and Kodak X-OMAT LS film (Eastman Kodak, Rochester, NY, USA). Quantitative data were obtained using ImageJ software (version 2.0, National Institutes of Health, Bethesda, MD, USA).

### 4.6. RNA Extraction and Quantitative Real-Time PCR (q-PCR)

Total RNA was extracted from glioma cells using TRIzol reagent (Invitrogen) and was quantified using the BioDrop spectrophotometer (Cambridge, UK). The target mRNA levels was detected using quantitative real-time PCR. The reverse transcription (RT) reaction was performed using 2 μg of total RNA converted into cDNA using the Invitrogen RT Kit and amplified using the following oligonucleotide primers: MGMT: 5′-CCTGGCTGAATGCCTATTTC-3′ and 5′-GATGAGGATGGGGACAGGATT-3′; Cx43: 5′-TCGGGTTAAGGGAAAGAG-3′ and 5′-GCTCACTTGCTTGCTTGT-3′; β-actin: 5′-AGAGCTACGAGCTGCCTGAC-3′ and 5′-AGCACTGTGTTGGCGTACAG-3′. The protocol of qPCR was referred to our previous report. PCR amplification was performed in final volumes of 10 μL using ExiLENT SYBR Green master mix (EXIQON).

### 4.7. Wound-Healing Assay

U251 or TMZ-resistant glioma cells were seeded onto cell culture plates with Ibidi Culture-Inserts in the middle of the wells. A cell-free gap of 500 μM was thus generated after removing the Ibidi Culture-Insert. The images were acquired after 0, 12, and 24 h using a digital camera and a light microscope.

### 4.8. Sulforhodamine B Assay (SRB)

The SRB assay is based on the measurement of cellular protein content and the procedure was followed according to our previous report [71]. After treatment with TMZ for 24 h, 48 h, and 72 h, cells were fixed with 10% trichloroacetic acid (TCA) and stained by SRB at 0.4% (w/v) in 1% acetic acid for 1 h. Unbound SRB was washed out by 1% acetic acid and SRB-bound cells were solubilized with 10 mM Trizma base. The absorbance was read at a wavelength of 515 nm using a microplate reader (Bio-Tek, Winooski, VT, USA).

### 4.9. PrestoBlue Proliferation Assay

U251 or TMZ-resistant glioma cells were seeded at a density of 5 × 10^3^ cells per well in 96 plates. The cells were cultured with medium containing 2% fetal bovine serum for 24 h, 48 h, and 72 h. The proliferation rate of these two cells was measured by using PrestoBlue cell viability reagent (Invitrogen, New York, NY, USA). According to the manufacturer’s protocol. The absorbance was read at a wavelength of 570/600 nm using a microplate reader (Bio-Tek, Winooski, VT, USA) after addition and incubation with the reagent.

### 4.10. Statistics

Statistical analysis was performed using GraphPad Prism6 software (version 6, GraphPad software Inc., San Diego, CA, USA) and SigmaPlot software (version 10.0, Systat Software Inc., San Jose, CA, USA). Data are presented as means ± S.E.M. Statistical analysis between two samples was assessed by a Student’s *t*-test. One-way ANOVA assessed statistical analysis between three or more independent group. In all cases, a value of *p* less than 0.05 was considered significant. *p*-Values are indicated in the figure legends. 

## 5. Conclusions

This study identified functional differences between the TMZ-resistant subline and the parental cells. Our results showed that connexin 43 was upregulated in the resistant U251 subline compared with the parental U251 cell. In addition, EMT-associated regulator molecules were downregulated in the resistant U251 subline. The resistant U251 subline also exhibited lower cell migration activity and monocyte adhesion ability compared with the parental U251 cell. Furthermore, the resistant U251 subline expressed lower levels of VCAM-1 after administration with the recombinant TNF-α. These findings suggested differential characteristics between the drug-resistant GBM and the parental glioma cells. These investigations demonstrated that the extent of intratumoral heterogeneity in glioma influences cell migratory activity as well as the expression of intrinsic adhesion proteins. Further functional studies on potential molecular markers may provide therapeutic strategies for targeting malignant glioma cells.
